# Progressive increases in adiposity and ectopic fat surrogates across glycaemic states highlight weight reduction as a key target for type 2 diabetes prevention, especially in younger people

**DOI:** 10.1111/dom.70248

**Published:** 2025-11-03

**Authors:** Sabrina Scilletta, Paul Welsh, Antonino Di Pino, Naveed Sattar

**Affiliations:** ^1^ School of Cardiovascular and Metabolic Health University of Glasgow Glasgow UK; ^2^ Department of Clinical and Experimental Medicine University of Catania Catania Italy

**Keywords:** glycaemic control, type 2 diabetes, weight control, weight management

## Abstract

**Aims:**

To determine whether there are progressive changes in weight and ectopic fat surrogates in individuals comparing normoglycaemia, prediabetes and undiagnosed type 2 diabetes, and whether these increments differ by age.

**Materials and Methods:**

Cross‐sectional analysis of UK Biobank White participants without baseline cardiovascular disease or known diabetes (*n* = 287 987). Participants were classified based on glycated haemoglobin (HbA1c): normal (<5.7% [38.9 mmol/mol]), prediabetes1 (5.7–5.9% [39.0–41.9 mmol/mol]), prediabetes2 (6%–6.2% [42.0–44.9 mmol/mol]), prediabetes3 (6.3%–6.4% [45–47.9 mmol/mol]) and undiagnosed diabetes (6.5%–9% [48–75 mmol/mol]). Ordered logistic and linear regressions were used to test associations between HbA1c groups and differences in body mass index (BMI), waist‐to‐height ratio (WHtR), alanine aminotransferase (ALT; a surrogate of liver fat with known limitations) and triglycerides (circulating fat), adjusting for age, sex, deprivation status and statin use. The impact of age was also considered.

**Results:**

BMI exhibited a stepwise increase across groups with the prediabetes1, prediabetes2, prediabetes3 and undiagnosed diabetes groups having 1.47, 2.86, 3.82 and 4.09 kg/m^2^ higher BMI, respectively, compared to the normal group; WHtR followed a similar trend, rising by 0.024, 0.047, 0.062 and 0.065 across groups, as did ALT levels by 2.45, 4.05, 6.11 and 10.28 U/L and triglycerides by 0.25, 0.42, 0.50 and 0.67 mmol/L, respectively. Such increments were greater in younger versus older people.

**Conclusions:**

Deteriorating glycaemic status was marked by progressively higher levels of adiposity and circulating and hepatic ectopic fat markers. Findings were more pronounced in younger individuals, suggesting a greater role for ectopic fat in their diabetes and reinforcing the importance of weight intervention for preventing the progression from normoglycaemia to prediabetes to frank diabetes.

## 1 INTRODUCTION

There is growing evidence that obesity and ectopic liver fat are the key drivers of type 2 diabetes.^1^ Epidemiological evidence strongly supports a causal relationship between obesity and diabetes, with high hazard and odds ratios observed. For example, men with severe obesity (body mass index [BMI] ≥ 35 kg/m^2^) have been shown to carry a substantially increased risk of developing diabetes compared to lean individuals, with relative risks exceeding 40 in some cohorts.^2^

Remission studies further support the causal role of obesity, showing a near‐linear relationship between the extent of weight loss and diabetes remission rates in people with recent‐onset diabetes.^3^, ^4^, ^5^ Both lifestyle interventions and pharmacotherapy have consistently demonstrated significant improvements in glycaemic control following weight loss.^6^, ^7^, ^8^

However, BMI alone does not fully capture individual metabolic risk.^9^ Ectopic fat accumulation—particularly in the liver—has been increasingly recognised as a mechanistic driver of type 2 diabetes.^10^, ^11^ Metabolic dysfunction‐associated steatotic liver disease (MASLD), a condition characterised by hepatic steatosis in the presence of metabolic risk factors, is present in most individuals with type 2 diabetes and is closely linked to metabolic dysfunction.^12^

Both MASLD and type 2 diabetes share a common driver in weight excess, and both conditions improve with intentional weight loss.^13^ In individuals with MASLD, changes in liver enzymes—particularly alanine aminotransferase (ALT)—have been shown to reflect reductions in liver fat following meaningful weight loss, offering a simple marker for clinical monitoring.^14^

These metabolic abnormalities are not limited to overt diabetes. Prediabetes—a state characterised by impaired fasting glucose or impaired glucose tolerance—also shows strong associations with adiposity and hepatic steatosis.^15^ Prediabetes affects nearly 300 million adults globally and is projected to rise to 414 million by 2045.^16^ Individuals with prediabetes have a ~50% risk of progressing to type 2 diabetes within 5 years.^17^ Importantly, interventions that target weight loss—through lifestyle changes, pharmacotherapy or bariatric surgery—have been shown to significantly reduce this risk and even prevent progression to diabetes.^18^, ^19^, ^20^, ^21^

While the role of excess adiposity and ectopic liver fat in established type 2 diabetes is well documented, less is known about how these parameters and associated weight differ across the entire glycaemic spectrum—from normoglycaemia to prediabetes and frank diabetes. Understanding these transitions in totality could help better emphasise the role of obesity in the development and progression of prediabetes and clarify whether these associations differ by age of development of prediabetes. The latter is important as younger people with type 2 diabetes, lose far more life years from diabetes,^22^ and mechanisms are not fully established, though hepatic insulin resistance and impaired beta‐cell function are likely to play important roles.

Capitalising on the availability of data in the UK Biobank comprising several hundred thousand participants, including glycated haemoglobin (HbA1c) and adiposity measures, this cross‐sectional study examined differences in adiposity measures and commonly measured surrogate markers of ectopic fat across the glycaemia life course. Specifically, this study examined BMI and waist‐to‐height ratio (WHtR) as markers of adiposity, and ALT (as a surrogate of hepatic fat) and triglycerides (as a circulating fat marker). It also uniquely stratified glycaemic status into finer HbA1c categories to test for stepwise changes in ectopic fat burden.

## 2 MATERIALS AND METHODS

The UK Biobank recruited >502 000 participants (age 37–73 years) from 22 assessment centres across the UK between April 2007 and December 2010. Baseline biological measurements were recorded and touch screen questionnaires were administered. The UK Biobank received ethics approval from the North West Multicenter Research Ethics Committee (reference number 11/NW/03820). All participants gave written informed consent before enrolment in the study, which was conducted in accordance with the principles of the Declaration of Helsinki. Systolic (SBP) and diastolic (DBP) blood pressure were taken as the first baseline measurement, preferentially using an automated measurement. Smoking status was categorised as never or former/current smoking. Ethnicity was coded as White, Black, South Asian or mixed/other, with White as the referent group. Blood collection sampling procedures for the study have previously been described and validated.^23^ Importantly, blood and urine samples were collected contemporaneously at the baseline assessment visit (2007–2010). These samples were subsequently stored and later assayed at a central laboratory between 2014 and 2017. These included plasma HbA1c (VARIANT II TURBO Haemoglobin Testing System; Bio‐Rad). Data were adjusted by UK Biobank centrally before release to adjust for preanalytical variables. Further details of these measurements, and of the data adjustments, can be found in the UK Biobank online showcase and protocol (http://www.ukbiobank.ac.uk and https://biobank.ndph.ox.ac.uk/showcase/showcase/docs/biomarker_issues.pdf). The definition of baseline diabetes included self‐reported type 1 or type 2 diabetes and self‐reported use of insulin. Statin and blood pressure medication use was also recorded from self‐report. Baseline cardiovascular disease (CVD) was defined as self‐reported prior myocardial infarction, stroke and transient ischemic attack as well as hospital diagnoses, including International Classification of Diseases, 10th Revision (ICD‐10) codes I20–24, I63–64 and G45. Participants with baseline CVD were excluded from all analyses, and those with baseline diabetes were also excluded. Non‐White participants were excluded.

### 2.1 Statistical methods

HbA1c was analysed as a continuous variable and was categorised using thresholds of 5.7% (38.9 mmol/mol) (normal/referent), 5.7%–5.9% (39.0–41.9 mmol/mol) (prediabetes1), 6%–6.2% (42.0–44.9 mmol/mol) (prediabetes2), 6.3%–6.4% (45.0–47.9 mmol/mol) (prediabetes3) and 6.5%–9% (48.0–75.0 mmol/mol) (undiagnosed diabetes). The upper limit for the undiagnosed diabetes group was set at 9% (75 mmol/mol) to minimise potential bias from unintentional weight loss due to glucosuria and catabolic effects that commonly occur at very high glycaemic levels.^24^ For ease of presentation and comparison, simple cut‐offs within the prediabetes HbA1c range were applied. These categories are not intended for clinical use, but should provide intuitive and easily interpretable groups that highlight the gradation of changes in adiposity and ectopic fat markers across small HbA1c increments. Classical CVD risk factors were expressed as mean (standard deviation [SD]) if symmetrically distributed, median (interquartile range) if skewed, and number (%) if categorical. Ordered logistic regression and linear regression were used to test associations between HbA1c groups and differences in BMI, waist‐to‐height ratio (WHtR), alanine aminotransferase (ALT; a surrogate of liver fat) and triglycerides (circulating fat), after adjusting for age, sex, deprivation status and statin use (as statins are known to influence HbA1c levels and potentially ALT levels), as these represent key demographic and clinical confounders most strongly associated with both adiposity and glycaemic status. Smoking, alcohol intake and physical activity (International Physical Activity Questionnaire [IPAQ]) were not included as covariates in the main models because these lifestyle factors may represent mediators rather than confounders of the relationship between adiposity and glycaemia. Participants were further stratified by age at recruitment, using the median age to define younger (≤57 years) and older (>57 years) subgroups. An interaction term between glycaemic group and age group was included to evaluate effect modification by age. Given the near‐balanced distribution of participants across the 22 assessment centres, and their role as administrative rather than sampling units, standard model‐based standard errors (SEs) were used. In this context, they are effectively equivalent to cluster‐robust SEs, with any residual centre‐level correlation expected to have negligible impact on inference. As a sensitivity analysis, all models were re‐estimated, including fixed effects for year of recruitment to account for potential secular trends; results were unchanged. All analyses were performed using STATA 14 (StataCorp). A two‐sided *p* value of 0.05 was considered statistically significant.

## 3 RESULTS

### 3.1 Population characteristics

Of 400 921 people without baseline CVD included in the study, complete data on covariates, including HbA1c, were available for 326 954 (81.5%) participants, and after exclusion of participants with known/self‐reported diabetes (*n* = 15 385), those with HbA1c >9% (75.0 mmol/mol) (*n* = 18 156), and non‐White participants (*n* = 15 422), the cohort for the main analyses included 265 664 participants (Figure S1). This also included individuals with HbA1c comprised between 6.5% and 9% (48.0 and 75.0 mmol/mol) without report of prior diabetes diagnosis (i.e., undiagnosed diabetes). Median HbA1c in this cohort was 5.4 ± 0.9% (35.7 ± 6.4 mmol/mol).

Participants were stratified into five groups according to HbA1c levels: normoglycaemia (≤5.7% [≤38.9 mmol/mol]; *n* = 232 109), prediabetes1 (5.7%–5.9% [39.0–41.9 mmol/mol]; *n* = 22 549), prediabetes2 (6%–6.2% [42.0–44.9 mmol/mol]; *n* = 6538), prediabetes3 (6.3%–6.4% [45.0–47.9 mmol/mol]; *n* = 2499) and undiagnosed diabetes (6.5%–9% [48.0–75.0 mmol/mol]; *n* = 1969).

Higher HbA1c levels were associated with older age and a higher male predominance relative to participants with normoglycaemic HbA1c (Table 1). Indicators of adiposity and ectopic fat burden were progressively higher across glycaemic categories. Mean BMI rose from 26.7 ± 4.3 kg/m^2^ in normoglycaemic participants to 31.4 ± 5.9 kg/m^2^ in those with undiagnosed diabetes, while WHtR increased from 0.52 ± 0.07 to 0.60 ± 0.09. A similar stepwise rise was observed in ALT, a surrogate marker of hepatic ectopic fat (22.8 ± 13.4 to 34.7 ± 22.7 U/L), and in circulating triglyceride levels (1.66 ± 0.96 to 2.43 ± 1.48 mmol/L).

**TABLE 1:** Population characteristics.

	Normal	PreD1	PreD2	PreD3	Undiagnosed diabetes	Total
	*N* = 232 109	*N* = 22 549	*N* = 6538	*N* = 2499	*N* = 1969	*N* = 265 664
Age at recruitment (years)	55.52 ± 8.13	59.67 ± 6.83*	60.48 ± 6.50*^#^	60.78 ± 6.43*^#^	58.18 ± 7.50*^#°†^	56.06 ± 8.11
Sex, *n* (%)						
Female	121 667 (52.4)	11 720 (52.0)	3081 (47.1)*^#^	1021 (40.9)*^#°^	702 (35.7)*^#°†^	138 191 (52.0)
Male	110 442 (47.6)	10 829 (48.0)	3457 (52.9)*^#^	1478 (59.1)*^#°^	1267 (64.3)*^#°†^	127 473 (48.0)
Weight (kg)	76.67 ± 14.99	80.49 ± 16.59*	85.13 ± 17.97*^#^	89.72 ± 18.44*^#^°	91.16 ± 19.07*^#°^	78.05 ± 15.94
BMI (kg/m^2^)	26.73 ± 4.27	28.38 ± 4.98*	29.99 ± 5.48*^#^	31.23 ± 5.57*^#°^	31.36 ± 5.93*^#°^	27.03 ± 4.48
WHtR	0.52 ± 0.07	0.55 ± 0.08*	0.58 ± 0.08*^#^	0.60 ± 0.08*^#°^	0.60 ± 0.09*^#°^	0.53 ± 0.07
Townsend deprivation index, *n* (%)						
Low deprivation	129 527 (55.8)	11 955 (53.0)*	3358 (51.4)*^#^	1203 (48.1)*^#°^	938 (47.6)*^#°^	146 981 (55.3)
Average deprivation	72 406 (31.2)	7224 (32.0)*	2061 (31.5)*^#^	813 (32.5)*^#°^	643 (32.7)*^#°^	83 147 (31.3)
Higher deprivation	30 176 (13.0)	3370 (14.9)*	1119 (17.1)*^#^	483 (19.3)*^#°^	388 (19.7)*^#°^	35 536 (13.4)
HDL cholesterol (mmol/L)	1.48 ± 0.38	1.40 ± 0.37*	1.31 ± 0.34*^#^	1.24 ± 0.32*^#°^	1.24 ± 0.34*^#°^	1.47 ± 0.38
LDL cholesterol (mmol/L)	3.59 ± 0.83	3.64 ± 0.93*	3.47 ± 0.97*^#^	3.17 ± 0.98*^#°^	3.38 ± 1.00*^#°†^	3.59 ± 0.85
Total cholesterol (mmol/L)	5.74 ± 1.08	5.78 ± 1.22*	5.53 ± 1.28*^#^	5.12 ± 1.30*^#°^	5.40 ± 1.29*^#°†^	5.73 ± 1.11
Triglycerides (mmol/L)	1.66 ± 0.96	1.93 ± 1.04*	2.13 ± 1.12*^#^	2.26 ± 1.21*^#°^	2.43 ± 1.48*^#°†^	1.71 ± 0.99
ALT (U/L)	22.75 ± 13.42	25.14 ± 14.47*	27.34 ± 15.39*^#^	30.23 ± 18.94*^#°^	34.66 ± 22.66*^#°†^	23.22 ± 13.80
CRP (mg/dL)	2.23 ± 3.84	3.19 ± 4.96*	3.97 ± 5.70*^#^	4.03 ± 5.80*^#^	4.64 ± 5.77*^#°†^	2.39 ± 4.06
Alcohol intake frequency, *n* (%)						
Daily or almost daily	53 117 (22.9)	4610 (20.4)*	1262 (19.3)*^#^	438 (17.5)*^#°^	383 (19.5)*^#^	59 810 (22.5)
Three or four times a week	60 363 (26.0)	4959 (22.0)*	1243 (19.0)*^#^	458 (18.3)*^#°^	376 (19.1)*^#^	67 399 (25.4)
Once or twice a week	61 058 (26.3)	5827 (25.8)*	1648 (25.2)*^#^	622 (24.9)*^#°^	498 (25.3)*^#^	69 653 (26.2)
One to three times a month	24 464 (10.5)	2709 (12.0)*	808 (12.4)*^#^	330 (13.2)*^#°^	247 (12.5)*^#^	28 558 (10.7)
Special occasions only	20 469 (8.8)	2670 (11.8)*	977 (14.9)*^#^	381 (15.2)*^#°^	281 (14.3)*^#^	24 778 (9.3)
Never	12 638 (5.4)	1774 (7.9)*	600 (9.2)*^#^	270 (10.8)*^#°^	184 (9.3)*^#^	15 466 (5.8)
Smoking status, *n* (%)						
Non‐smoker	130 684 (56.3)	10 778 (47.8)*	2824 (43.2)*^#^	1053 (42.1)*^#°^	844 (42.9)*^#^	146 183 (55.0)
Ex‐smoker	80 744 (34.8)	8538 (37.9)*	2681 (41.0)*^#^	1097 (43.9)*^#°^	829 (42.1)*^#^	93 889 (35.3)
Smoker	20 681 (8.9)	3233 (14.3)*	1033 (15.8)*^#^	349 (14.0)*^#°^	296 (15.0)*^#^	25 592 (9.6)
Statin use, *n* (%)	26 171 (11.3)	5872 (26.0)*	2572 (39.3)*^#^	1396 (55.9)*^#°^	786 (39.9)*^#†^	36 797 (13.9)
SBP (mmHg)	136.67 ± 18.45	140.74 ± 18.57*	142.67 ± 18.37*^#^	143.63 ± 18.27*^#^	144.82 ± 18.52*^#°†^	137.29 ± 18.54
DBP (mmHg)	82.03 ± 10.12	82.91 ± 10.17*	83.41 ± 10.02*^#^	82.98 ± 10.20*	84.37 ± 11.07*^#°†^	82.17 ± 10.14
IPAQ activity group, *n* (%)						
Sedentary	40 552 (17.5)	4288 (19.0)*	1451 (22.2)*^#^	634 (25.4)*^#°^	558 (28.3)*^#°†^	47 483 (17.9)
Moderate	94 690 (40.8)	9256 (41.0)*	2639 (40.4)*^#^	1007 (40.3)*^#°^	774 (39.3)*^#°†^	108 366 (40.8)
Active	96 867 (41.7)	9005 (39.9)*	2448 (37.4)*^#^	858 (34.3)*^#°^	637 (32.4)*^#°†^	109 815 (41.3)

*Note*: Values are presented as mean ± standard deviation (SD) for continuous variables and as number (percentage) for categorical variables. Glycaemic status was categorised as: normal (glycated haemoglobin [HbA1c] ≤5.7% [≤38.9 mmol/mol]), PreD1 (prediabetes1, HbA1c 5.7%–5.9% [39.0–41.9 mmol/mol]), PreD2 (prediabetes2, HbA1c 6%–6.2% [42.0–44.9 mmol/mol]), PreD3 (prediabetes3, HbA1c 6.3%–6.4% [45.0–47.9 mmol/mol]) and undiagnosed diabetes (HbA1c 6.5%–9% [48.0–75.0 mmol/mol]). Significance levels: *p < 0.05 versus normal; ^#^ *p* < 0.05 versus PreD1;°*p* < 0.05 versus PreD2; ^†^ *p* < 0.05 versus PreD3 (Bonferroni‐adjusted for continuous variables; *χ* ^2^ test for categorical variables).

Abbreviations: ALT, alanine aminotransferase; BMI, body mass index; CRP, C‐reactive protein; DBP, diastolic blood pressure; HDL, high‐density lipoprotein; IPAQ, International Physical Activity Questionnaire; LDL, low‐density lipoprotein; SBP, systolic blood pressure; WHtR, waist‐to‐height ratio.

Across increasing HbA1c strata, high‐density lipoprotein (HDL) cholesterol levels declined, while C‐reactive protein (CRP) concentrations exhibited a progressive increase. Participants with prediabetes and undiagnosed diabetes were also more likely to be more socioeconomically deprived, current smokers, sedentary, and to use statins compared to those with normal HbA1c. These differences were even more pronounced among individuals with undiagnosed diabetes, particularly BMI, WHtR, triglycerides and markers of inflammation.

### 3.2 Associations between glycaemic categories and adiposity/ectopic fat markers

**FIGURE 1:**
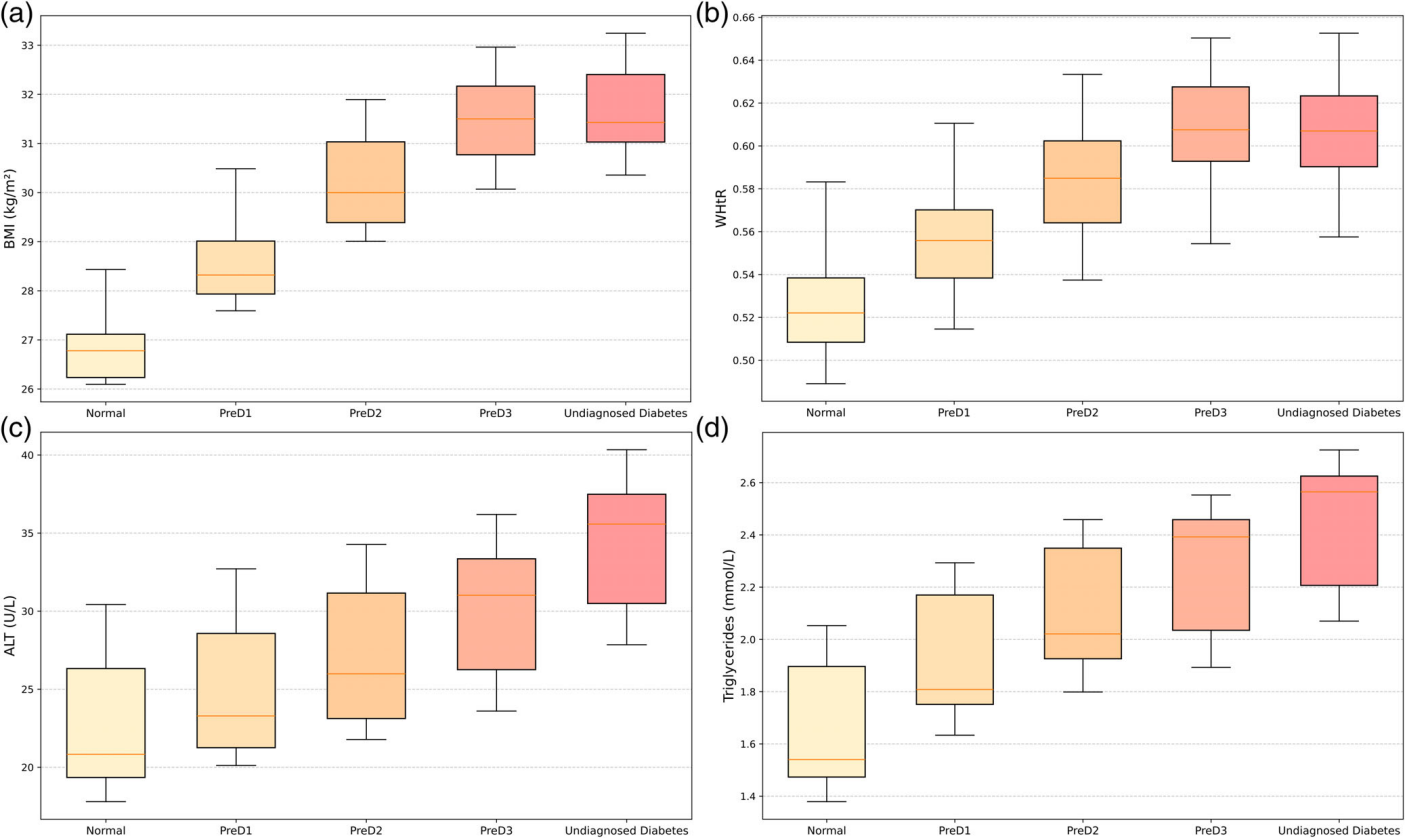
Body mass index (BMI), waist‐to‐height ratio (WHtR), alanine aminotransferase (ALT) and triglycerides across glycated haemoglobin (HbA1c) groups. Adjusted mean values (±95% confidence intervals) of metabolic markers across glycaemic categories: (A) BMI, (B) WHtR, (C) ALT and (D) triglycerides. All markers show a progressive increase with worsening glycaemic status after adjustment for age, sex, deprivation index and statin use (all *p* < 0.001). Glycaemic status was categorised as follows: normal: HbA1c ≤5.7% (≤38.9 mmol/mol), PreD1: HbA1c 5.7%–5.9% (39.0–41.9 mmol/mol), PreD2: HbA1c 6.0%–6.2% (42.0–44.9 mmol/mol), PreD3: HbA1c 6.3%–6.4% (45.0–47.9 mmol/mol) and undiagnosed diabetes: HbA1c 6.5%–9.0% (48.0–75.0 mmol/mol). Associations were tested using linear regression models adjusted for age, sex, socioeconomic deprivation status and statin use.

After adjustment for age, sex, deprivation index and statin use, all adiposity and ectopic fat markers were significantly associated with worsening glycaemic status in a stepwise fashion (as shown in Figure 1). Compared with individuals in the normoglycaemic group, adjusted mean BMI was higher by 1.47 (95% confidence interval 1.41–1.53), 2.86 (2.75–2.97), 3.82 (3.64–3.99) and 4.09 (3.90–4.28) kg/m^2^ in the prediabetes1, prediabetes2, prediabetes3 and undiagnosed diabetes groups, respectively. These BMI increases correspond to estimated average body weight differences of approximately 8–12 kg, which increase progressively across categories.

Similarly, WHtR increased across groups by 0.024 (0.023–0.025), 0.047 (0.045–0.048), 0.062 (0.059–0.065) and 0.065 (0.062–0.068), respectively. Increases were also observed in circulating and hepatic fat markers: ALT rose by 2.45 (2.27–2.64), 4.05 (3.73–4.38), 6.11 (5.59–6.64) and 10.28 (9.69–10.86) U/L across the prediabetes1 and undiagnosed diabetes groups, while triglycerides were higher by 0.25 (0.23–0.26), 0.42 (0.39–0.44), 0.50 (0.46–0.54) and 0.67 (0.63–0.72) mmol/L, respectively, in comparison to the normoglycaemic group. Each transition in HbA1c category was associated with a statistically significant increase in these biomarkers, reinforcing the broad linearity of the association between adiposity, ectopic fat and worsening glycaemic control (all *p* < 0.001; see Table 2).

**TABLE 2:** Stepwise increases in adiposity and ectopic fat markers across glycaemic categories.

	Normal	PreD1	PreD2	PreD3	Undiagnosed diabetes
Weight (kg)	Referent	+3.98* [3.83, 4.12]	+7.75* [7.49, 8.01]	+10.92* [10.49, 11.34]	+11.73* [11.26, 12.21]
BMI (kg/m^2^)	Referent	+1.47* [1.41, 1.53]	+2.86* [2.75, 2.97]	+3.82* [3.64, 3.99]	+4.09* [3.90, 4.28]
WHtR	Referent	+0.024* [0.023, 0.025]	+0.047* [0.045, 0.048]	+0.062* [0.059, 0.065]	+0.065* [0.062, 0.068]
ALT (U/L)	Referent	+2.45* [2.27, 2.64]	+4.05* [3.73, 4.38]	+6.11* [5.59, 6.64]	+10.28* [9.69, 10.86]
Triglycerides (mmol/L)	Referent	+0.25* [0.23, 0.26]	+0.42* [0.39, 0.44]	+0.50* [0.46, 0.54]	+0.67* [0.63, 0.72]

*Note*: Glycaemic status was categorised as: normal (glycated haemoglobin [HbA1c] ≤5.7% [≤38.9 mmol/mol]), PreD1 (prediabetes1, HbA1c 5.7%–5.9% [39.0–41.9 mmol/mol]), PreD2 (prediabetes2, HbA1c 6%–6.2% [42.0–44.9 mmol/mol]), PreD3 (prediabetes3, HbA1c 6.3%–6.4% [45.0–47.9 mmol/mol]) and undiagnosed diabetes (HbA1c 6.5%–9% [48.0–75.0 mmol/mol]). All markers were adjusted for age, sex, deprivation index and statin use. Values in bold and marked with * indicate statistically significant differences (*p* < 0.05) versus the preceding group.

Abbreviations: ALT, alanine aminotransferase; BMI, body mass index; WHtR, waist‐to‐height ratio.

In sensitivity analyses additionally adjusting for smoking status, physical activity (IPAQ) and alcohol intake, the results were materially unchanged (Figure S2).

### 3.3 Age‐stratified trends of adiposity/ectopic fat markers across glycaemic categories

**FIGURE 2:**
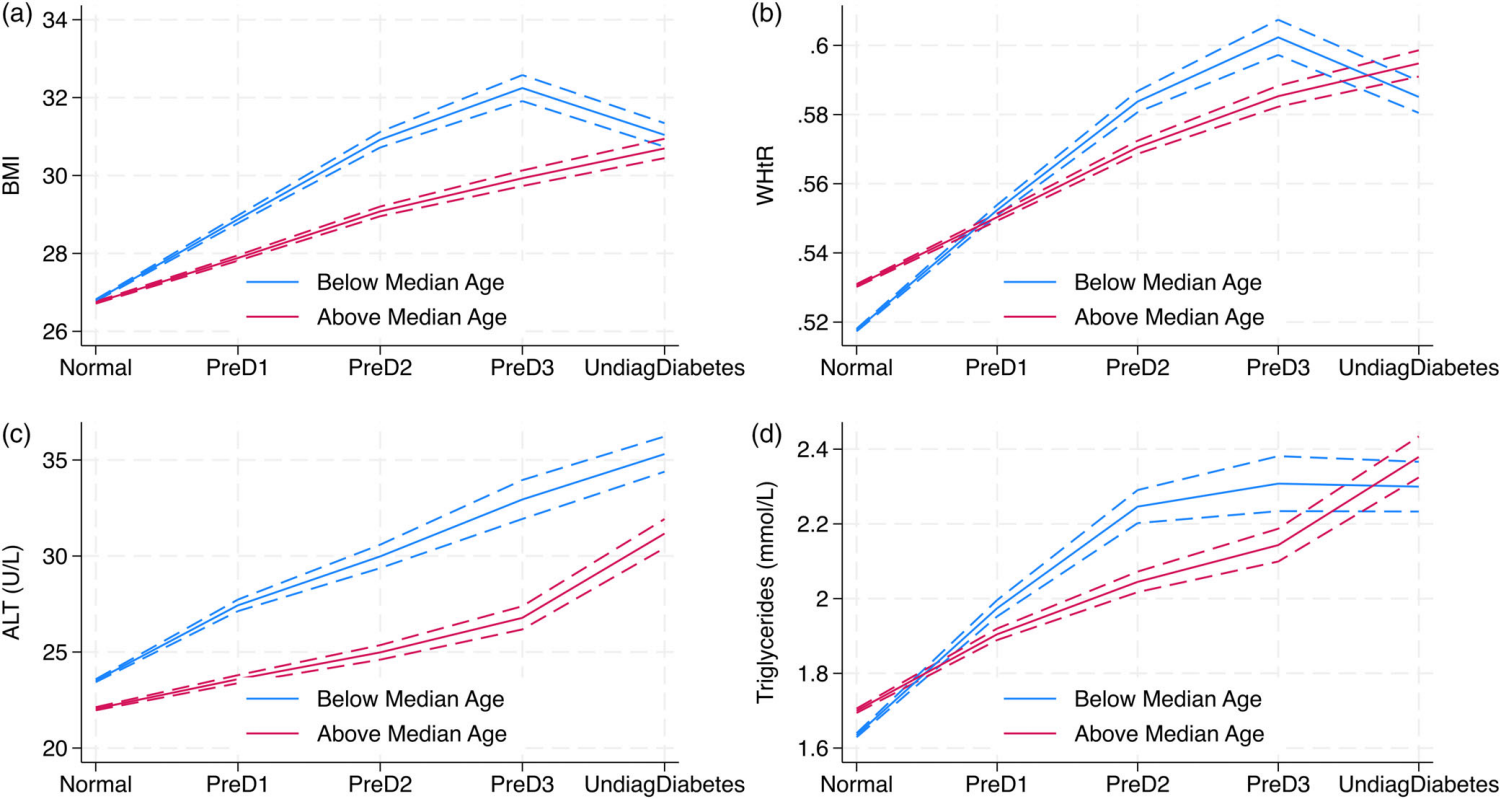
Age‐stratified trends in body mass index (BMI), waist‐height ratio (WHtR), alanine aminotransferase (ALT) and triglycerides by glycaemic status. Younger individuals consistently show higher BMI (panel A), WHtR (panel B), ALT (panel C) and triglycerides (panel D) adjusted levels across all glycaemic stages, with more pronounced differences in the prediabetic phases. Predicted means (with 95% confidence intervals [CI]) of BMI, WHtR, ALT and triglyceride levels across increasing glycaemic categories, stratified by median age. Glycaemic status was categorised as: normal (glycated haemoglobin [HbA1c] ≤5.7% [≤38.9 mmol/mol]), PreD1 (prediabetes1, HbA1c 5.7%–5.9% [39.0–41.9 mmol/mol]), PreD2 (prediabetes2, HbA1c 6%–6.2% [42.0–44.9 mmol/mol]), PreD3 (prediabetes3, HbA1c 6.3%–6.4% [45.0–47.9 mmol/mol]) and undiagnosed diabetes (undiagnosed diabetes, HbA1c 6.5%–9% [48.0–75.0 mmol/mol]). Lines represent mean values with 95% CI for individuals below (blue) and above (purple) the median age. Associations were estimated using linear regression models adjusted for age, sex, socioeconomic deprivation status and statin use. A significant overall interaction between glycaemic status and age group (above vs. below median age) was observed (*p*‐interaction <0.0001) for all four measures.

As shown in Figure 2, the association between glycaemic status and markers of adiposity and ectopic fat was more pronounced among younger individuals (aged ≤57 years). Compared with normoglycaemic participants in the same age group, those in the prediabetes1, prediabetes2 and prediabetes3 groups exhibited substantially greater increases in adjusted mean BMI, WHtR, ALT and triglyceride levels. For example, among younger participants, the adjusted mean BMI was higher relative to normoglycaemic peers by 2.15 kg/m^2^ in prediabetes1, 4.17 kg/m^2^ in prediabetes2 and 5.74 kg/m^2^ in prediabetes3. In contrast, the corresponding BMI differences in older individuals were somewhat lower: 1.16, 2.42 and 3.32 kg/m^2^, respectively.

Similarly, increases in WHtR and triglycerides were more accentuated in the younger group across prediabetes stages, although the age‐related difference was less apparent in the undiagnosed diabetes category. Notably, ALT increased progressively with worsening glycaemic status across all categories in younger individuals, and the absolute differences in ALT values between glycaemic categories were consistently greater than those observed in older individuals, including in the group with undiagnosed diabetes (Figure 2). These findings were supported by a highly significant overall glycaemic status × age interaction (*p*‐interaction <0.0001).

These findings were consistent when using alternative age strata (18–44, 45–64 and ≥65 years), as shown in Figure S3.

## 4 DISCUSSION

In this large cohort of over 260 000 individuals spanning a broad glycaemic spectrum—from normoglycaemia through various stages of prediabetes to undiagnosed type 2 diabetes—we observed a corresponding stepwise progression in markers of adiposity and ectopic fat with increasing HbA1c levels. Each transition in HbA1c category was associated with a statistically significant difference in these adjusted biomarkers, reinforcing the broad linearity of the association between adiposity (as measured by BMI or WHtR), ectopic fat (ALT) and circulating fat (triglycerides) with progressively worsening glycaemic control.

Our findings reinforce and extend important *holistic* evidence on the relationship between adiposity, ectopic fat surrogates and dysglycaemia across *all* clinical thresholds. While the association between obesity and type 2 diabetes is well established—with a meta‐analysis reporting an approximately seven‐fold higher risk in individuals with obesity compared to those of normal weight^25^—data specifically addressing the difference below and beyond the prediabetic state remains comparatively limited.

In a cohort of over 100 000 Chinese adults, a clear dose–response relationship between BMI and prediabetes risk was observed, with elevated risk evident even within the normal BMI range (≥23.03 kg/m^2^), indicating that adverse metabolic effects of adiposity may manifest earlier than previously appreciated.^26^ Similarly, the large Jingchang prospective cohort (*n* > 48 000) demonstrated positive associations between both BMI and WHtR and the risk of impaired fasting glucose.^27^ Concordant findings have also been reported in South Asian populations.^28^ Our results confirm and meaningfully expand upon these observations in a distinct (White‐only) and larger population, showing a stepwise increase in both BMI and WHtR across worsening HbA1c categories. They also show what average change of BMI and weight is roughly needed to revert from prediabetes to normoglycaemia, important given the availability of more tools by which to aid intentional weight loss. As such, we believe these data will help many more doctors better appreciate that type 2 diabetes and its precursor of prediabetes, are both intimately linked to gains in ectopic fat, such that large‐scale weight loss at diabetes diagnosis could help reversion to normoglycaemia in many, as recently reviewed.^29^

The relationship between ectopic fat, prediabetes and type 2 diabetes has previously been studied,^30^ though it is in a limited fashion. For example, findings from the Azar cohort study in Iran, including 15 000 adults, demonstrated a strong association between elevated hepatic enzymes and both prediabetes and diabetes; serum levels of ALT were significantly higher among individuals with prediabetes and diabetes compared to normoglycaemic individuals. Importantly, a dose–response relationship was observed across quartiles of these enzymes, with progressively higher odds ratios for both prediabetes and diabetes from the lowest to the highest quartile of ALT.^31^ Our findings, which show these relationships in, what we believe to be, a much more intuitive manner by showing a progressive increase in ALT levels across worsening glycaemic categories, extend these observations. In turn, our findings support the role of ectopic hepatic fat accumulation as an early marker of metabolic dysregulation, with potential implications for risk stratification well before overt diabetes onset.

In addition to adiposity and ectopic fat, our findings highlight a strong, progressive association between glycaemic deterioration and circulating triglyceride levels. This is consistent with recent evidence from a large systematic review and meta‐analysis by Hu et al.,^32^ which identified elevated triglyceride levels as a significant clinical risk factor for the progression from prediabetes to type 2 diabetes. Their findings, based on 59 cohort studies, reinforce the notion that dyslipidaemia—particularly elevated triglycerides—parallels worsening insulin sensitivity and metabolic control in prediabetic individuals. This makes sense as circulating triglyceride when elevated most often relates to excess ectopic fat.^33^

Finally, our findings indicate that the metabolic burden associated with dysglycaemia is markedly amplified in younger individuals, who exhibit steeper increases in markers of adiposity (BMI and WHtR), and most notably, liver fat (ALT), and circulating triglycerides across glycaemic categories. These data, perhaps especially the higher ALT levels, suggest that younger individuals require a greater degree of metabolic/ectopic fat stress—particularly in terms of weight gain—to manifest prediabetes or diabetes. This aligns with previous studies showing that younger adults diagnosed with type 2 diabetes exhibit larger differences in BMI and other metabolic parameters compared to their age‐matched normoglycaemic individuals, as opposed to older adults at diagnosis, where such differences are attenuated.^34^ In that study, individuals diagnosed with diabetes at age 20–39 had an average weight excess of ~18.7 kg compared to their non‐diabetic peers, while those diagnosed at age ≥80 had a difference of only ~5.3 kg. These findings reinforce the importance of early detection and prevention strategies tailored to younger adults in whom more rapid weight gain is a major concern with multiple ‘upstream’ drivers.^35^

Our results have practical implications for clinical practice. They broadly support the notion that effective weight management in Whites, and particularly in younger people, will play a crucial role in preventing the progression from normoglycaemia to prediabetes, and eventually to type 2 diabetes and its complications. With an expanding arsenal of tools—including lifestyle interventions, pharmacological therapies and bariatric surgery—effective weight management will be more accessible to individuals living with obesity to reduce the risk of glycaemic deterioration and its detrimental impact on health and quality of life. Intentional weight loss can also help people become more active, to further improve glycaemia status.

As with any study, our work has strengths and limitations. The current study is strengthened by its large sample size, detailed phenotyping and inclusion of a broad glycaemic spectrum from normoglycaemia to undiagnosed diabetes, enabling a nuanced analysis of adiposity and ectopic fat markers across progressive HbA1c categories. Rather than introducing a completely novel concept, this work builds on established evidence and provides robust quantification and detailed mapping within a single large cohort. Furthermore, the use of routinely available and clinically relevant, easily measurable markers—BMI, WHtR, ALT and triglycerides—enhances the translational applicability of our findings to real‐world clinical settings. However, the study also has limitations. The UK Biobank cohort exhibits a healthy volunteer bias, with participants generally healthier than the broader UK population, potentially limiting the generalisability of findings to the general population. In particular, we restricted analyses to White participants to reduce heterogeneity and increase internal validity, given known higher prevalences of prediabetes and diabetes at lower BMIs in many non‐Whites for differential reasons (e.g., more ectopic fat or lower beta cell function) that have the potential to mask meaningful associations. In addition, sample sizes for other ethnic groups in UK Biobank were limited, making subgroup analyses underpowered and prone to spurious findings. We recognise this as a limitation in generalisability and strongly support efforts to improve representation and power for ethnicity‐stratified analyses in future research. Our aim was to establish robust baseline patterns within a more homogeneous reference group, from which future studies can build using more diverse cohorts. Moreover, while ALT is a widely used surrogate marker for hepatic steatosis, it can also be influenced by other factors such as hepatic inflammation, alcohol intake, medications (including statins) and subclinical liver conditions, which may partially confound its interpretation as a direct proxy for liver fat. Similarly, triglyceride levels are not specific to ectopic fat linked to obesity, as they can be elevated due to alcohol, some medications and genetic predispositions. Nevertheless, when changes in weight, ALT and triglycerides occur in parallel, this strengthens the interpretation of changes in ectopic fat burden. Furthermore, the cross‐sectional nature of the study precludes causal inference regarding the observed associations, though prior studies strongly suggest a causal association of liver fat with type 2 diabetes.^36^ Finally, HbA1c as an outcome also has limitations. HbA1c levels can be influenced by factors beyond glycaemia, such as severe anaemia, differences in kidney function and certain haemoglobin variants. Although HbA1c is widely used in both clinical practice and epidemiological research as a robust marker of average glycaemia, these factors may sometimes introduce variability that is not directly related to glucose homeostasis.

In conclusion, these analyses provide robust evidence of a stepwise association between worsening glycaemic status and higher levels of adiposity and in circulating and hepatic ectopic fat markers, with such increments being greater in younger people with prediabetes and diabetes, in keeping with their need for greater adiposity to develop type 2 diabetes. These findings reinforce weight intervention programmes as a primary intervention target for preventing the progression from normoglycaemia to prediabetes to frank type 2 diabetes, largely via reduction of ectopic fat, as well as for large‐scale weight loss to enable remission from early type 2 diabetes to normoglycaemia.

## AUTHOR CONTRIBUTIONS

NS and SS conceived the idea and wrote the first draft. PW and ADP critically reviewed and edited the manuscript. All authors approved the final version of the manuscript.

## FUNDING INFORMATION

The authors declare no funding.

## CONFLICT OF INTEREST STATEMENT

Paul Welsh reports grant income from Roche Diagnostics, AstraZeneca, Boehringer Ingelheim and Novartis, and speaker fees from Novo Nordisk and Raisio outside the submitted work. Naveed Sattar has consulted for and/or received speaker honoraria from Abbott Laboratories, AbbVie, Amgen, AstraZeneca, Boehringer Ingelheim, Carmot Therapeutics, Eli Lilly, GlaxoSmithKline, Hanmi Pharmaceuticals, Menarini‐Ricerche, Metsera, Novartis, Novo Nordisk, Pfizer and Roche; and received grant support paid to his University from AstraZeneca, Boehringer Ingelheim, Novartis and Roche outside the submitted work. Sabrina Scilletta and Antonino Di Pino have no conflicts to declare.

## Supporting information


**Data S1.** Supporting Information.

## Data Availability

Source data can be requested from the UK Biobank. Analytic codes are available upon request.
