# Supplementary material for: Progressive increases in adiposity and ectopic fat surrogates across glycaemic states highlight weight reduction as a key target for type 2 diabetes prevention, especially in younger people
Source: Diabetes Obes Metab. 2025 Nov 3;28(1):654–62. doi: 10.1111/dom.70248 (PMC12673443; doi:10.1111/dom.70248)
Supplement: Supplementary file 1 — Data S1. Supporting Information. [file DOM-28-654-s001.pdf]

Supplementary Materials

Progressive Increases in Adiposity and Ectopic Fat Surrogates Across Glycemic States Highlight Weight Reduction as a Key Target for Type 2 Diabetes Prevention, especially in younger people

Sabrina Scilletta[1,2], Paul Welsh[1], Antonino Di Pino[2], Naveed Sattar[1]

1 School of Cardiovascular and Metabolic Health, University of Glasgow, Glasgow, Scotland

2 Department of Clinical and Experimental Medicine, University of Catania, Catania, Italy

Supplementary Figure 1: Flow chart of the study participants

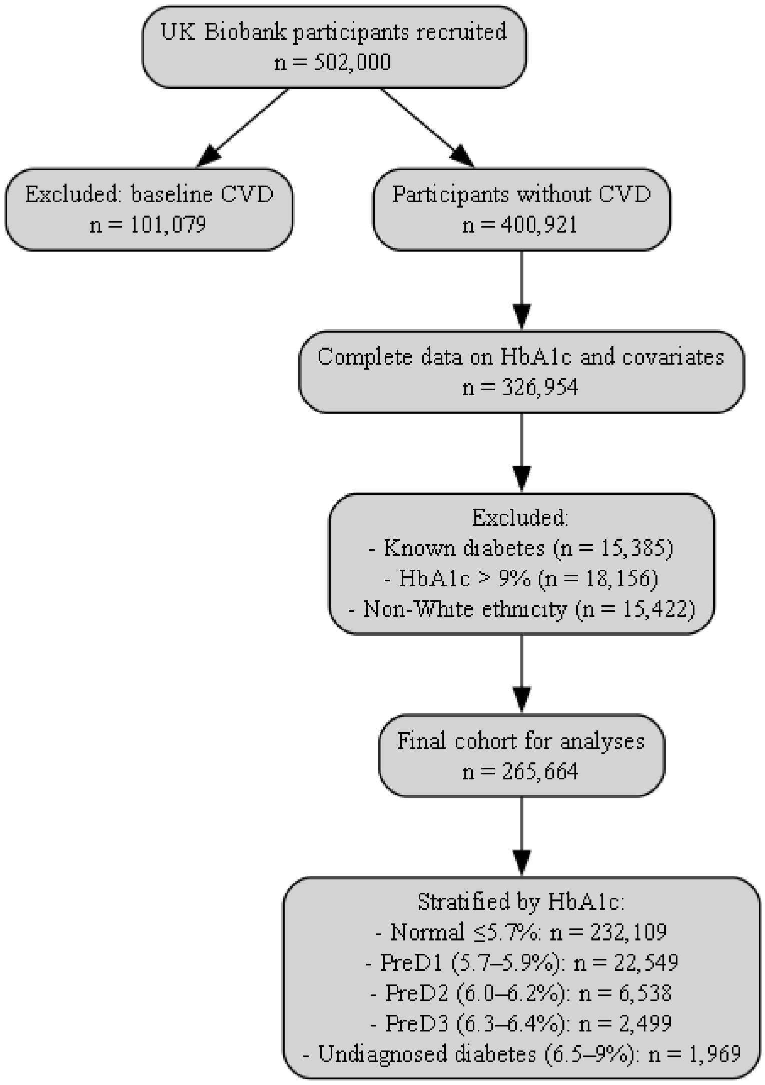

Glycemic status was categorized as: normal (HBA1c ≤5.7% [≤38.9 mmol/mol]), PreD1 (prediabetes1, HBA1c 5.7-5.9% [39.0–41.9 mmol/mol]), PreD2 (prediabetes2, HBA1c 6-6.2% [42.0–44.9 mmol/mol]), PreD3 (prediabetes3, HBA1c 6.3-6.4% [45.0–47.9 mmol/mol]), and undiagnosed diabetes (undiagnosed diabetes, HBA1c 6.5-9% [48.0–75.0 mmol/mol]). CVD: Cardiovascular disease.

Supplementary Figure 2: BMI, WHtR, ALT and triglycerides across HbA1c groups.

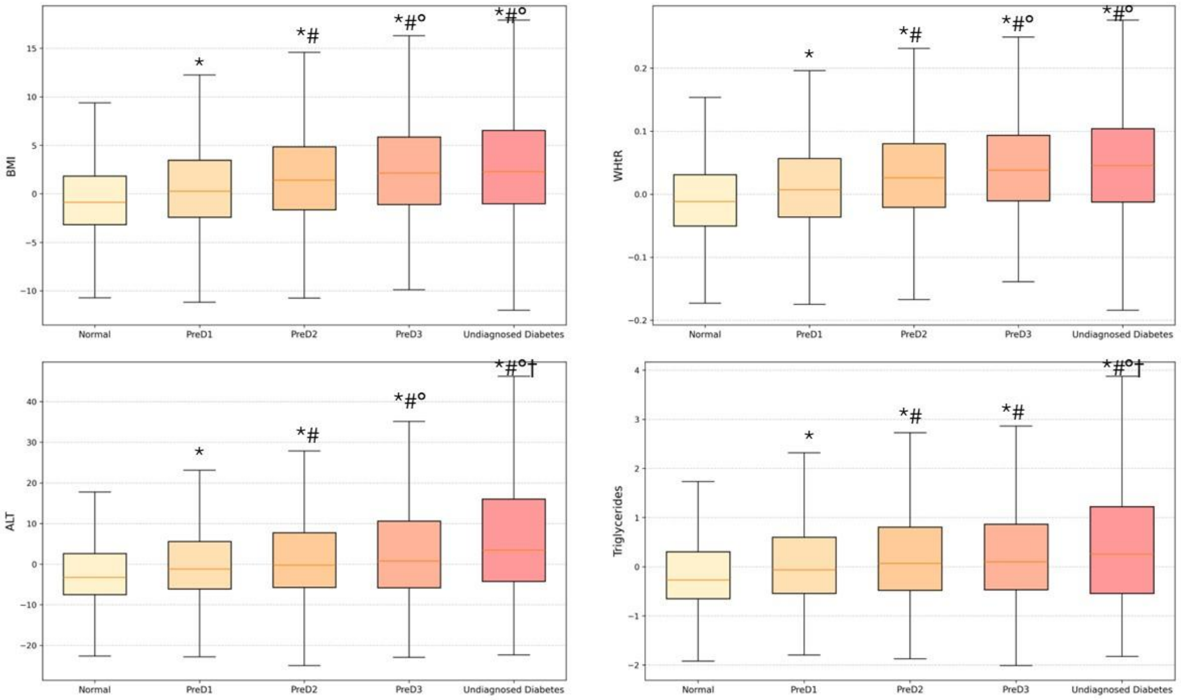

Adjusted mean values (±95% CI) of metabolic markers across glycemic categories: (A) Body mass index (BMI), (B) Waist-to-height ratio (WHtR), (C) Alanine aminotransferase (ALT), and (D) Triglycerides. Glycemic status was categorized as follows: Normal: HbA1c ≤5.7% (≤38.9 mmol/mol), PreD1: HbA1c 5.7–5.9% (39.0–41.9 mmol/mol), PreD2: HbA1c 6.0–6.2% (42.0–44.9 mmol/mol), PreD3: HbA1c 6.3–6.4% (45.0–47.9 mmol/mol), and Undiagnosed diabetes: HbA1c 6.5–9.0% (48.0–75.0 mmol/mol). *p < 0.05 vs. Normal; #p < 0.05 vs. PreD1; °p < 0.05 vs. PreD2; †p < 0.05 vs. PreD3. Significance levels are Bonferroni-adjusted for continuous variables. BMI: Body mass index; WHtR: Waist-to-height ratio; ALT: alanine aminotransferase; IPAQ: International Physical Activity Questionnaire. Models adjusted for age, sex, socioeconomic deprivation status, statin use, physical activity (IPAQ), alcohol intake, and smoking status.

Supplementary Figure 3: Age-stratified trends in BMI, WHtR, ALT, and triglycerides by glycemic status.

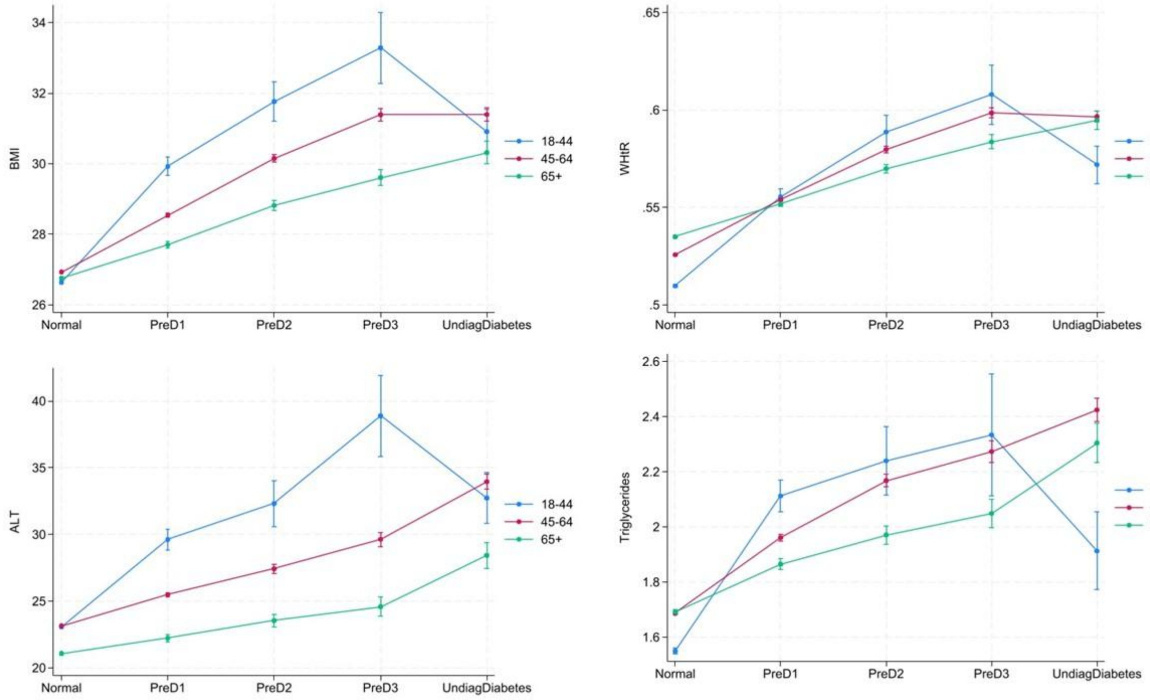

Adjusted mean values (±95% CI) of metabolic markers across HbA1c categories within age groups: (A) Body mass index (BMI), (B) Waist-to-height ratio (WHtR), (C) Alanine aminotransferase (ALT), and (D) Triglycerides. Participants were stratified into three age groups: 18–44 years, 45–64 years, and ≥65 years. Glycemic status was categorized as: Normal: HbA1c ≤5.7% (≤38.9 mmol/mol), PreD1: HbA1c 5.7–5.9% (39.0–41.9 mmol/mol), PreD2: HbA1c 6.0–6.2% (42.0–44.9 mmol/mol), PreD3: HbA1c 6.3–6.4% (45.0–47.9 mmol/mol), and Undiagnosed diabetes: HbA1c 6.5–9.0% (48.0–75.0 mmol/mol). Models were adjusted for age, sex, deprivation status, and statin use.
